# Hypertrophic neovaginal granulation tissue after gender affirming vaginoplasty: A cautionary tale and novel treatment option

**DOI:** 10.1016/j.eucr.2025.103070

**Published:** 2025-05-16

**Authors:** Gaia Sartorelli, Marissa Kent

**Affiliations:** a Tufts University School of Medicine, 145 Harrison Ave, Boston, MA, 02111, USA; b Beth Israel Deaconess Medical Center, 330 Brookline Ave, Boston, MA, 02215, USA

**Keywords:** Gender-affirming vaginoplasty, Hypertrophic granulation tissue, Neovaginal complications, Holmium laser

## Abstract

Approximately 25 million people worldwide, including 1 million in the United States, identify as transgender. Gender affirmation surgery (GAS), including vaginoplasty, improves quality of life but carries a 20–70 % complication rate. Granulation tissue occurs in 26 % of cases, but treatment for severe cases remains unclear. We present one transfeminine individual with extensive neovaginal granulation tissue involving 70 % of the neovagina which was subsequently treated with laser destruction. This case illustrates a novel management strategy and underscores the need for standardized treatment approaches for severe granulation tissue following GAS.

## 1 Introduction

Gender affirming care consists of a multi-faceted and inter-disciplinary approach that incorporates angles such as social transitioning, psychological support, hormone therapy and gender affirming surgery (GAS). For some individuals, the pursuit of gender affirming genital surgery is associated with lower levels of depression and alleviation of gender dysphoria.^1^^,^^2^ Vaginoplasty for transfeminine individuals, utilizing the penile inversion or peritoneal pull through method, is a type of feminizing GAS that is associated with high levels of satisfaction.^3^ Several complications are associated with vaginoplasty including wound dehiscence, tissue necrosis, neovaginal stenosis, divergent urinary stream, and rectal or urethral injury.^1^^,^^2^ Of the various post-operative complications, granulation tissue is one of the most common with an overall rate of occurrence of up to 26 %.^3^ Currently there is no evidence-based guideline on the management of hypergranulation tissue in this setting.^4^ The literature is limited to individual surgeon experiences, primarily addressing external granulation tissue, with no direct focus on the consequences of these treatments in the deeper vaginal canal.^4^, ^5^, ^6^

This case report describes extensive granulation tissue in the vaginal canal of a transfeminine patient post-peritoneal pull through vaginoplasty, highlighting potential complications of repeated treatment and proposing a novel alternative.

## 2 Case presentation

**Fig. 1:**
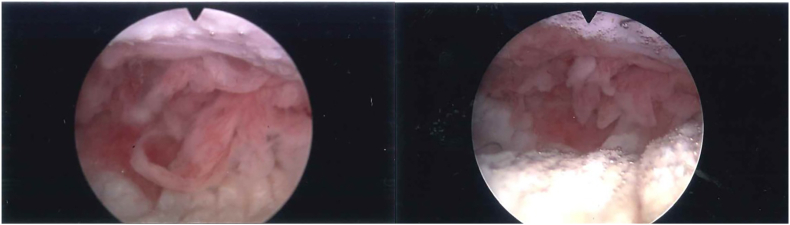
Flexible cystoscope was used to visualize frondular tissue in the posterior vagina with pathology consistent with granulation tissue.

The patient is a 23 year-old transgender female who presented as a referral for pain with dilations 3 months after peritoneal pull through vaginoplasty. Her surgery was performed in May 2021 in Thailand by another surgeon. On an office speculum exam, she had friable tissue lining the vaginal walls with purulent discharge. Culture of the discharge returned mixed bacterial flora. She was treated for vaginitis with topical metronidazole and was advised to increase her douching regimen. At her follow up visit a month later, the purulent discharge had resolved but she was still experiencing difficulty with initial dilator insertion and bleeding with dilations. Office vaginoscopy with a flexible cystoscope revealed a scar tissue bridge 3 cm inside the introitus and marked frondular tissue in the posterior vagina, with a canal depth of 9.5 cm [Fig. 1]. An office biopsy of the tissue confirmed granulation tissue. Given the skin bridge and extensive granulation tissue with fronds, the decision was made to bring her to the operating room for excision of the skin bridge, holmium laser destruction of the granulation tissue, and dilation. The holmium laser destruction was performed using a resectoscope with the laser bridge attached. The fronds were destroyed at the base. At the end of the procedure, vaginal packing and a foley catheter were placed and then removed in the office after 48 hours. In follow-up, now 28 months post procedure, she has maintained functional vaginal depth without any pain or bleeding.

## 3 Discussion

Granulation tissue is a common complication after gender affirming vaginoplasty, occurring in up to 26 % of individuals, yet no evidence-based guidelines exist for its management. Current approaches are largely extrapolated from management of granulation tissue in other areas of the body or individual surgeon experience with treatment in these vaginoplasty patients.^4^ Our case presents a novel use of holmium laser for treating deep vaginal granulation tissue. To our knowledge this is the first published paper outlining the use of holmium lasers for this purpose. Over two years post treatment, the results have been durable for the patient presented in our case. A second patient, a 31 year-old transgender female, developed severe vaginal stenosis following repeated treatments for refractory granulation tissue after penile inversion vaginoplasty. Multiple silver nitrate applications and curettage lead to extensive scarring, ultimately resulting in loss of vaginal depth. While this case is not the primary focus of this report, it illustrates what can happen when hypergranulation tissue in the deep vaginal canal is repeatedly treated with silver nitrate and electrocautery. This cautionary tale demonstrates the great need for additional treatment methods which can prevent scarring and loss of vaginal depth.

Hypertrophic granulation tissue is characterized by an excessive growth of granulation tissue during the proliferative stage of healing which then prevents maturation and epithelialization. Younger vaginoplasty patients often have many established risk factors for hypertrophic granulation tissue in other areas of the body including external friction from repeated dilations, increased bacterial microbiome, moisture, and low oxygen exposure.^4^ When found in the neovaginal canal, granulation tissue can have a red color that is either flat or may have raised projections of tissue that stand out. Granulation tissue lacks pain receptors, but in vaginoplasty patients, impaired healing, friable tissue, and superimposed infection can cause bleeding, pain and dilation difficulties. In some this may also lead to loss of vaginal dimensions due to less frequent dilations or the granulation tissue occupying space in the canal.^4^^,^^6^

This past year Potter and colleagues published a paper outlining the available literature on management of hypertrophic granulation tissue in the neovagina post vaginoplasty in addition to expert opinion.^4^ The paper discusses management strategies, including risk factor modification by using a hypertonic douching solution, maintaining dryness, and continued dilation.^4^ Treatment options include application of silver nitrate, topical steroids and surgical excision.^4^ Though not mentioned in his paper, cautery ablation is another treatment but risks stenosis. Secondary infections of hypertrophic granulation tissue are also common and it has been established that the neovagina in vaginoplasty patients has a high bacterial microbiome.^6^^,^^7^ For these reasons, we believe it is important to assess for and treat any potential superinfection to help manage risk factors prior to initiating any topical steroids. For patients with extensive hypertrophic granulation tissue, like the patient we present here, alternative treatments are limited when conservative measures fail, and excision risks compromising vaginal dimensions, highlighting the need for innovation.

The holmium laser, used in urology since the 1980s, is highly absorbed by water, with surface tissue penetration limited to 0.5 mm. This allows for precise incisions with only a small area being degraded by the heat.^8^ We performed the procedure by using a continuous flow resectoscope with the laser bridge attachment. This allows for the vaginal canal to distend with a continuous flow and the magnification of the scope allows for better visualization. When ablating the granulation tissue projections, we focused on ablating the base which proved to be effective.

## 4 Conclusion

This case emphasizes the importance of a personalized approach when managing post-vaginoplasty complications. Each patient's goals, symptoms severity, and anatomical considerations are crucial in guiding treatment options. In cases of severe or persistent granulation tissue formation, more aggressive interventions, including cautery, laser destruction, or surgical revision, may be necessary to achieve symptom relief and functional outcomes.

With this case, we aim to contribute to the growing body of literature on management of post-vaginoplasty complications, specifically hypertrophic neovaginal granulation tissue. Endoscopic laser destruction may be an effective therapeutic option for managing extensive granulation tissue by offering symptom relief and maintaining vaginal canal patency. Future research is needed to further define optimal treatment protocols for this complication particularly in the context of individualized care for transgender women undergoing gender-affirming surgery.

## CRediT authorship contribution statement

**Gaia Sartorelli:** Investigation, Project administration, Resources, Writing – original draft, Writing – review & editing. **Marissa Kent:** Conceptualization, Investigation, Methodology, Resources, Supervision, Writing – original draft, Writing – review & editing.

## Funding

Funding for publication fee provided by Beth Israel Deaconess Medical Center, Department of Urology.
